# Novel patch biomaterial treatment for colon diverticulosis in swine model

**DOI:** 10.3389/fbioe.2023.1215362

**Published:** 2023-07-28

**Authors:** Xiaomei Guo, Bhavesh Patel, Ling Han, William G. Van Alstine, Jillian N. Noblet, Sean D. Chambers, Ghassan S. Kassab

**Affiliations:** ^1^ California Medical Innovations Institute Inc, San Diego, CA, United States; ^2^ Cook Research Incorporated, West Lafayette, IN, United States; ^3^ Cook Medical, Bloomington, IN, United States

**Keywords:** colon diverticula, PVP patch, treatment, safety, pig model

## Abstract

Current leading managements for diverticular disease cannot prevent the recurrence of diverticulitis, bleeding and/or other complications. There is an immediate need for developing new minimal invasive therapeutic strategies to prevent and treat this disease. Through a biomechanical analysis of porcine colon with diverticular lesions, we proposed a novel adhesive patch concept aiming at mechanical reconstruction of the diseased colon wall. This study aims to evaluate the surgical feasibility (safety and efficacy) of pulmonary visceral pleura (PVP) patch therapy using a pig model of diverticulosis. Six female Yucatan miniature pigs underwent collagenase injection (CI) for the development of diverticular lesions. The lesions in each animal either received patch implantation (treated group, *n* = 40 for 6 pigs) or left intact (untreated group, *n* = 44 for 6 pigs). The normal colonic wall in each animal received patch implantation at two spots to serve as control (n = 12 for 6 pigs). After 3 months of observation, the performance and safety of the patch treatment were evaluated through macroscopic and histological examination. We found that 95% of pouch-like herniation of the mucosa was prevented from the colon wall with the treatment. The pouch diameter was significantly reduced in the treated group as compared to the untreated group (*p* < 0.001). The patch application caused a significant increase in the levels of collagen of the colon tissue as compared to the untreated and control groups (*p* < 0.001). No difference was found in the lymphocyte and macrophage inflammatory infiltrate between the groups. Our results suggest that patch treatment efficiently inhibits the diverticular pouch deformation and promotes the healing of the colon wall with a normal inflammatory response, which may minimize the risk of diverticulosis reoccurrence and complications over time.

## Introduction

Diverticular disease is highly prevalent in Western countries, but its incidence has been gradually increasing in the entire world over the past decade (Fong et al., 2011; Alatise et al., 2012; Isohata et al., 2021). The risk of acquiring the disease rises uniformly with age, with approximately 50% of people aged more than 60 years being affected in developed countries (Peery et al., 2016; Strate and Morris, 2019). Although most people with colonic diverticulosis remain asymptomatic, about 10%–25% of patients will have an attack of diverticulitis during their lifetime (Tursi et al., 2015; Reitano et al., 2023). This disease is becoming the leading cause of lower gastrointestinal bleeding in the United States (∼30% of the cases) (Ghassemi and Jensen, 2013), and one of the most common reasons for elective colon resection (Hawkins et al., 2020). Although fiber, antibiotics and probiotics seem to be effective in treating symptomatic and uncomplicated patients (Elisei and Tursi, 2016; Tochigi et al., 2017), the overall recurrence rate following the conservative treatments was reported to be as high as 28% (Perrone et al., 2021). Resection of the infected portion of the colon is currently the mainstay of therapy for chronic complications of diverticular disease. Elective colon resection, however, is related to a high rate of anastomotic bleeding or leakage or other postoperative complications (Kirchhoff et al., 2010; Wu et al., 2017). Specifically, post-surgery development of diverticula still occurred in certain patients (Giulio et al., 2022; Waser et al., 2023). Hence, there is a substantial desire for developing new therapeutic strategies to reduce the chance of recurrence of diverticulosis and its complications.

Utilization of clinically relevant large animal models of diverticulosis is essential for advancing our understanding of diverticulosis pathogenesis and developing new therapies, especially with minimally invasive surgical interventions (Patel et al., 2018). It is well known that collagen is the most abundant protein in mammals and serves to maintain the structural integrity of connective tissues (Lord et al., 1977). By creating a weakness in the colon wall with collagen fiber degradation, we have developed the first large animal model to replicate the pathology of diverticulosis in swine (Guo et al., 2019). Based on the biomechanical analysis of porcine colon with diverticular lesions (Patel et al., 2020), we proposed an adhesive patch concept aiming at mechanical reconstruction of the diseased colon wall. The major rationale is that an increase in local stress will cause progressive dilation and growth of colon tissue that can result in diverticulum while reinforcement of the tissue with an external patch will reduce the stress on the diverticulum and allow for reverse remodeling and healing of the colon tissue. Our group recently identified that pulmonary visceral pleura (PVP) from bovine may be a suitable biomaterial for the patch treatment of diverticulosis due to its beneficial mechanical and structural features (Lu et al., 2019; Lu et al., 2022). We hypothesize that application of a PVP patch would reduce the deformation of diverticula pouch and mitigate the natural progression of diverticulosis over time. The goal of this study is to evaluate the surgical feasibility (safety and efficacy) of adhesive PVP patch therapy using our pig model of diverticulosis.

## Materials and methods

### Experimental animals

Six female Yucatan miniature pigs at ages 2–8 years old were used in the study. All pigs underwent collagenase injection (CI) for 3 months followed by a 3-month patch treatment. Of the 84 diverticular lesions developed in 6 pigs at 3 months following CI, 40 were randomly selected to receive adhesive patch implantation as the treated group for 3 months, and the remaining 44 were left intact as the untreated group for 3 months. For each pig, the normal colonic wall (without diverticular lesions) also received patch treatment at 2 regions to serve as control (*n* = 12 for 6 pigs). All animal experiments were performed in accordance with national and local ethical guidelines, including the Institute of Laboratory Animal Research guidelines, Public Health Service policy, the Animal Welfare Act, as approved by Institutional Animal Care and Use Committee at California Medical Innovations Institute, San Diego.

### Collagenase injection

Collagenase injection (CI)-induced diverticulosis in swine was described in detail in our previous publication (Guo et al., 2019). In brief, pigs were fasted for 12 h prior to surgery. Pigs were pre-anesthetized with TKX (Telazol 10 mg/kg, Ketamine 5 mg/kg, and Xylazine 5 mg/kg, i.m.). Pigs were then anesthetized with 2%–3% isoflurane inhalation. A laparotomy (midline incision) was performed, and the descending colon was exposed gently. Collagenase (10u/μL, 200 µL) was injected into the colonic wall at 10–15 spots along the descending colon (the length of the treated colon segment is around 15–20 cm). The injected spots started to bleed due to collagen digestion in the colon 20 min following the injection. Electrocautery was then applied to stop bleeding. After the colon was inspected for bleeding, the abdominal incision was closed.

### PVP patch preparation

The PVP was peeled from fresh bovine lungs (provided by a local slaughterhouse) with hydro-dissection as described in our previous publication (Lu et al., 2021). The PVP patch was then fixed in 0.65% glutaraldehyde for overnight and stored in 0.25% glutaraldehyde. All patches were sterilized with a solution containing 2.05 g/L NaOH, 10.83 g/L PO4H2K, 200 mL/L Alcohol, 40 mL/L 25% glutaraldehyde, 110 mL/L 4% formaldehyde at 37°C for 24 h and then thoroughly rinsed in saline before *in vivo* implantation.

### Adhesive PVP treatment

**FIGURE 1:**
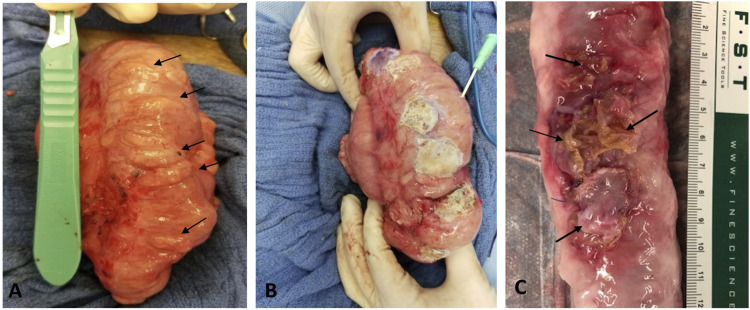
**(A)** *In vivo* image of diverticular lesions (arrows) following 3 months of collagen injection. **(B)** *In vivo* image of the lesions covered with adhesive PVP patch during the surgical patch implantation. A catheter was inserted into lumen to deflate the colon before the patch placement. **(C)** *Ex vivo* image of treated spots (arrows) after 3 months of treatment.

Pigs were fasted for 12 h prior to the procedure at 3 months following CI. Under anesthesia with TKX and followed by 2%–3% isoflurane inhalation, a laparotomy (midline incision) was performed. The descending colon was exposed gently, and diverticular pouch-like lesions (up to 20 mm in maximum diameter) were found and measured in the CI-treated colon segment (Figure 1A). On a normal colon spot (no diverticular lesions), an 18-gauge catheter was inserted into the colon lumen. Since air in the colon was released from the catheter, the intracolonic pressure was dropped, and the diverticular pouch was deflated. The PVP patch was trimmed to a size slightly larger than the lesion. Histoacryl (a tissue adhesive, around 200–300 µL) was applied evenly over the surface of the diverticular lesions. The PVP patch was then placed on top of the histoacryl layer to cover the lesion area. After the adhesive was allowed to harden for 30–60 s, the patch was firmly attached to the colon and prevented the diverticular pouch from protruding out of the colon wall (Figure 1B). The abdominal incision was closed after inspection.

### Terminal study

At the end of 3-month patch treatment, animal was anesthetized, and the abdomen was opened. The descending colon was exposed and macroscopically inspected for the appearance of PVP patch, changes of diverticular lesions (e.g., extent of pouch protrusion), signs of focal inflammation (e.g., erosions, erythema, edema and hyperemia) and presence of complications (e.g., bleeding, colonic rigidity, stenosis and adhesion). Autonomic intestinal peristalsis in treated colon segments was also observed and recorded. The animal was then euthanized with a saturated solution of potassium chloride (120 mL) injection through the jugular vein to arrest the heart under deep anesthesia. The descending colon was harvested for histological analysis.

### Histological assessment

The histological assessment of patch treatment included three aspects: 1) Dimensional changes of diverticular lesions (by measuring pouch opening size); 2) Morphological changes of the colon wall; 3) Colonic inflammatory response. The colon tissue was fixed with 4% paraformaldehyde for 48 h. The tissue was embedded in paraffin and sectioned at 4 µm thickness. Section was then processed with standard Hematoxylin and Eosin (H&E) and Trichrome staining for comprehensive overview. To exhibit the microscopical changes of the full-thickness colon wall structure, multiphoton microscope (MPM, Zeiss LSM 710 NLO) was used to scan the representative frozen tissue sections. Briefly, the paraformaldehyde-fixed colon tissue was embedded in OCT medium and frozen sectioned at 7 um thickness. The sections were incubated with standard fluorescent dyes (Phalloidin, Alexa 488 and Hoechst 33342) and imaged by MPM. For visualization and quantitation of collagen, the colon section was stained with Picro Sirius red. Six randomly selected microscopic fields (10X) on the diseased site were imaged for each section. The quantitative estimation of collagen content was conducted by ImageJ software (NIH) (Chen et al., 2017). The severity of inflammatory response was assessed by a semi-quantitation of lymphocytes and macrophages count. An immunofluorescence detection of lymphocyte with an anti-CD3 antibody and macrophage with an anti-CD68 antibody was performed, and the average score for cell count of the randomly chosen five microscopic fields (40X) on each section was calculated for CD3 and CD68. The inflammatory cells infiltration was graded according to a scale of 0–3 as listed in Table 1.

**TABLE 1:** The grading standard and scores of lymphocyte (CD3) and macrophage (CD68) inflammatory infiltrate in control, untreated and treated groups after 3 months of treatment on magnification (×40).

Grading standard:
0 to 3 cells: normal infiltrate (score 0)
4 to 6 cells: mild (score 1)
7 to 10 cells: moderate (score 2)
>10 cells: severe (score: 3)

Average Scores for 3 groups:			
	Control	Untreated	**Treated**
CD3	1.26 ± 0.30	1.06 ± 0.22	1.13 ± 0.17
CD68	1.23 ± 0.30	1.33 ± 0.20	1.11 ± 0.28

Values are means ± SE.

### Statistical analysis

Data was expressed as mean ± SD or mean ± SE as specified. The significance of the differences between treated and untreated groups was evaluated by X^2^-test or One-way ANOVA. The results were considered statistically significant when *p* < 0.05 (2-tailed). Statistical analysis was performed using SigmaStat (Version 4.0, Systat Software Inc, California, USA).

## Results

All experimental pigs subjected to CI and patch treatment survived until the end of the study. After the initial surgical procedure, pigs were monitored daily for appetite, general body condition, behavior, mobility and defecation until termination. All animals showed tolerance to the surgical interventions throughout the follow-up period and maintained their weights with no significant change before and after patch treatment (110 ± 11.6 kg vs. 115 ± 12.9 kg). No animal developed signs of infection, bleeding, constipation, bowel obstruction, or other postoperative complications.

**FIGURE 2:**
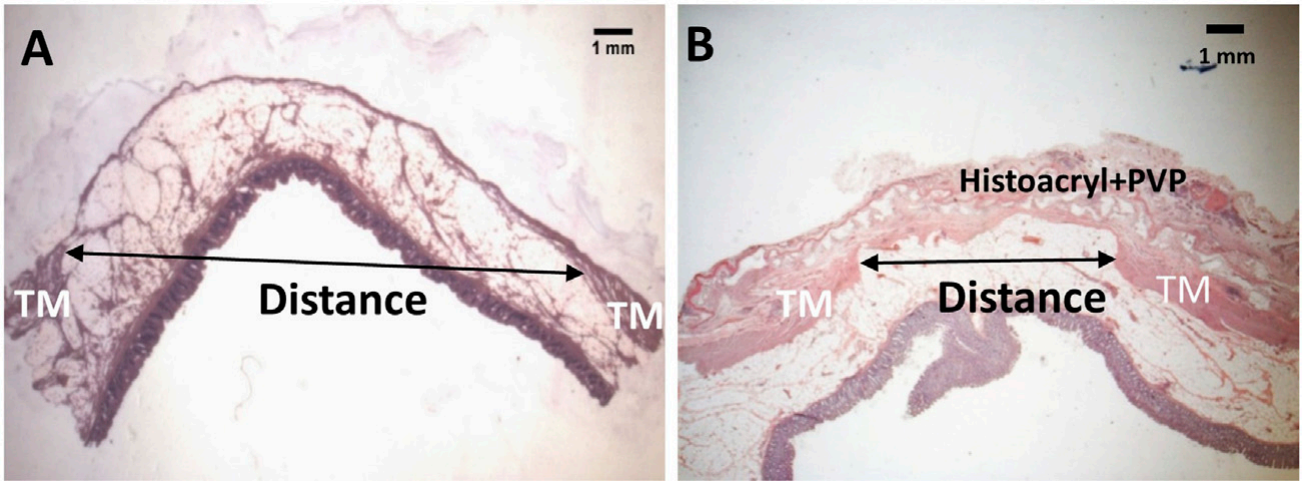
A schematic outline of the measurement of diameter for a diverticulum **(A)** and an adhesive patch-treated diverticulum **(B)** with H&E staining. TM: tunic muscularis.

Three months following CI, a total of 84 lesions (defined as mucosa herniation ≥ 2 mm aborally from the colon wall) were identified macroscopically from all 6 pigs (Figure 1A). Upon termination after 3 month-patch treatment for 40 lesions, we found that around 95% (38/40) of the PVP patches remained adhered to the original application sites and prevented mucosa protrusion (diverticular pouches) as shown in Figure 1C. Partial or complete implant dislocation/detachment occurred in 2 treated lesions (5%) causing persistent mucosa protrusion. For those untreated lesions (*n* = 44), all diverticular pouches persisted throughout the study. The patch significantly reduced the rate of pouch protrusion compared to the untreated sites (38/40 vs. 0/44, X^2^ test, *p* < 0.001). To assess the dimensional changes of the lesions in response to the treatment, the diameter of the diverticular pouch (defined as the distance of muscle separation) was measured using H&E histological images as schematically shown in Figure 2. The pouch diameter was significantly decreased in the treated lesions as compared to the untreated lesions (11.1 ± 2.6 mm vs. 6.6 ± 1.8 mm, *p* < 0.001).

During the surgical patch implantation, we noticed that 3 animals (50%) had focal intra-abdominal adhesion at 1-2 spots between the large intestine caused by CI-induced diverticula formation. Those adhesions are regarded as mild because they were dissectible with dull dissection. Three months after patch treatment, no remarkable changes in the levels and locations of the adhesions were seen and no new focal adhesion was formed. During the terminal study, we also observed an autonomic intestinal peristalsis in treated colon segments with no visible signs of rigidity and stenosis in all 6 pigs.

**FIGURE 3:**
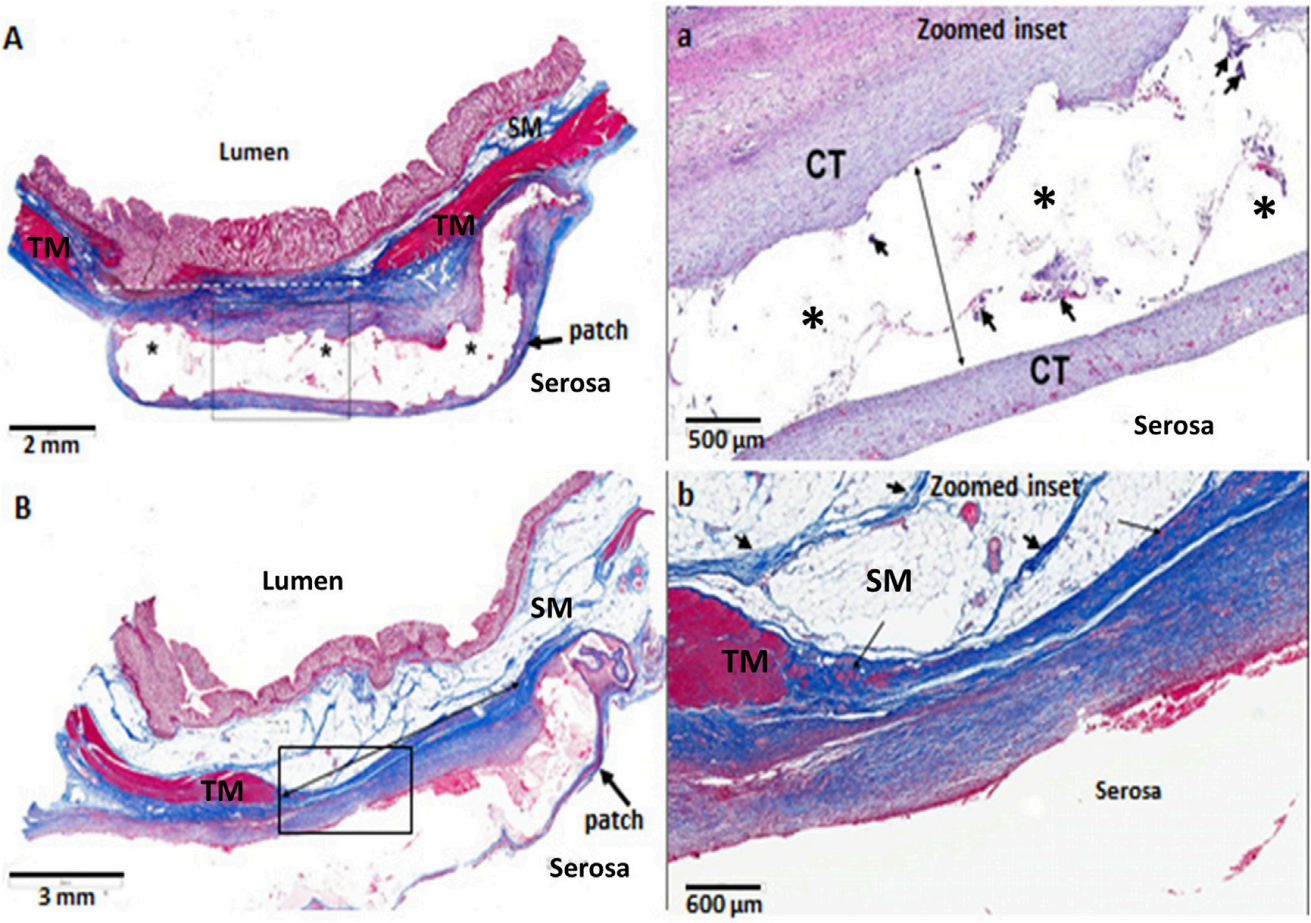
Histological images of diverticular lesions **(A,B)** following 3 months of adhesive PVP patch treatment (Trichrome staining). **(A)** The tunic muscularis (TM, red) within the injury area (dotted line) was replaced by connective tissue (blue) and covered with PVP patch (*). The submucosa (SM) is thinned and contains increased connective tissue. At higher magnification of the box in A **(a)**, the clear space (* and long arrow) is where the treatment material was placed. The area contains a translucent adhesive, patch, minimal connective tissue and low numbers of multinucleated macrophages (arrowheads). The PVP patch is surrounded by mature connective tissue (CT). **(B)** The TM (red) within the injury area (solid line) was replaced by connective tissue (blue) and covered with PVP patch. At higher magnification of the box in B **(b)**, connective tissue (blue) surrounds the serosal surface, which contains occasional clusters of smooth muscle cells (arrows). The SM is composed of normal fat and strands of fibrous connective tissue (arrowheads).

**FIGURE 4:**
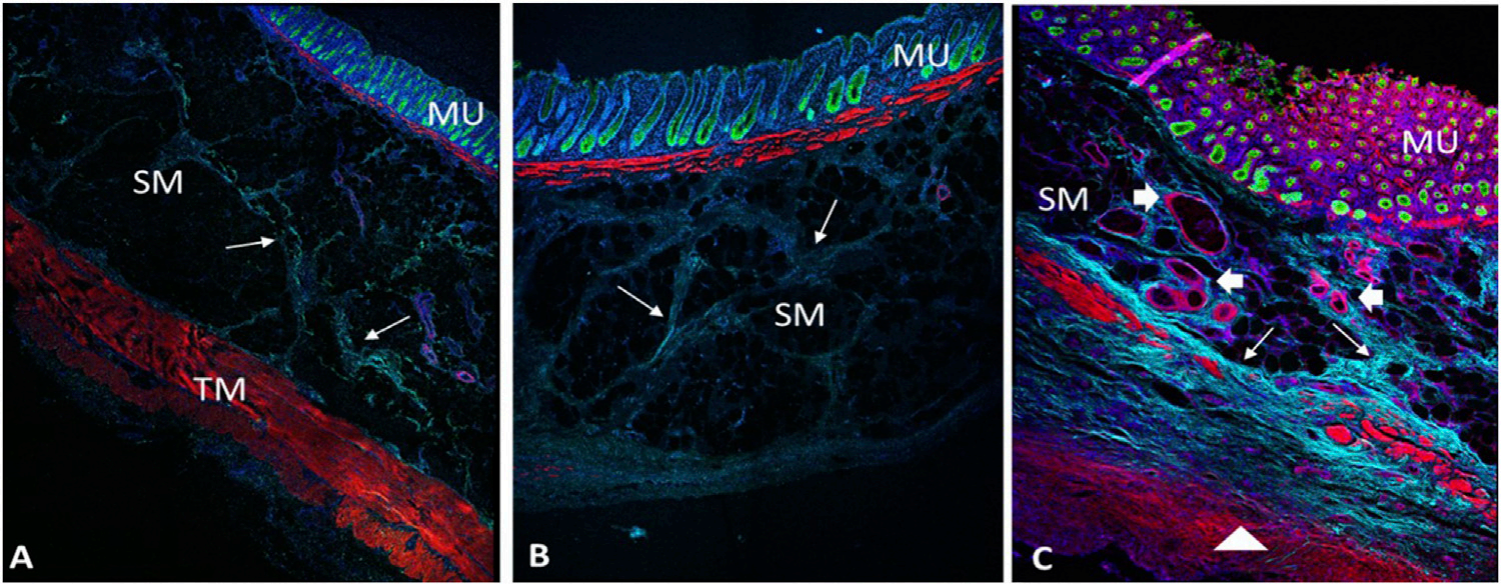
Multiphoton microscope images of control **(A)**, untreated **(B)** and treated diverticulum **(C)** with Immunofluorescence staining. TM, tunic muscularis; SM, submucosa; MU, mucosa. Red, smooth muscle F-actin stained with Phalloidin; Green, mucosa stained with Alexa 488; Light blue, collagen autofluorescence; Dark blue, nuclei stained with Hoechst 33342. **B**, an untreated diverticulum shows lack of tunic muscularis layer with slight collagen deposition (arrows). **C**, a treated diverticulum shows absence of TM layer, but with newly growing disorganized muscle cells (triangle), more collagen deposition (arrows) and vessels regeneration (arrowheads).

**FIGURE 5:**
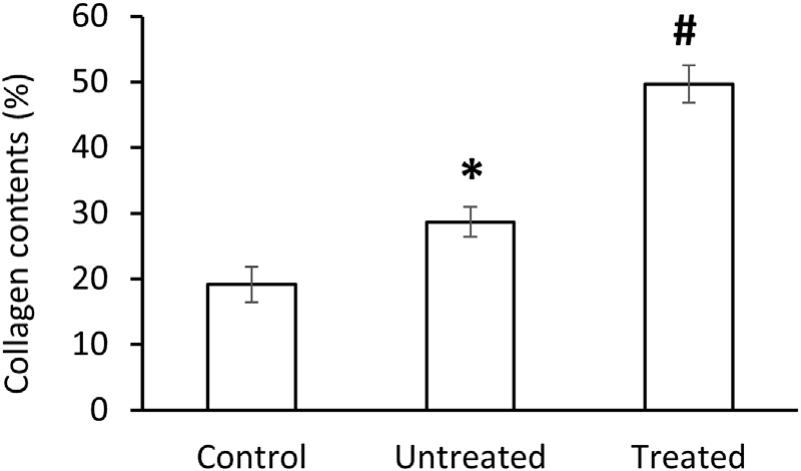
Collagen contents in control, untreated and treated groups. Data corresponds to mean ± SE. *p < 0.05, when compared to control group, ^#^ *p* < 0.05, when compared to untreated and control groups.

The morphology of the diverticular lesions with patch treatment was assessed by Trichrome staining (Figure 3). Although some of the implant materials were washed out during the histological process, the area of application was clearly detectable under a microscope. The diseased colon exhibited focal loss of tunic muscularis which was replaced by the remnant adhesive and PVP patches in combination with varying degrees of connective tissue, cellular debris and scattered lymphocytes and multinucleated macrophages. Multiphoton microscope images of tissue sections clearly displayed that tissue repair on the treated lesions was exuberant with increased collagen formation and appearance of aberrant bundles of smooth muscle cells (SMCs) (Figure 4). With Picro Sirius red staining, deposition of collagen fibers was seen within the submucosa layer of the colon wall (pictures are not shown). Quantitative analysis found that the collagen content was significantly higher in the treated group than that in the untreated and control groups (*p* < 0.01, Figure 5). The increased collagen content was also found in the untreated group when compared to the control group (*p* < 0.01, Figure 5).

**FIGURE 6:**
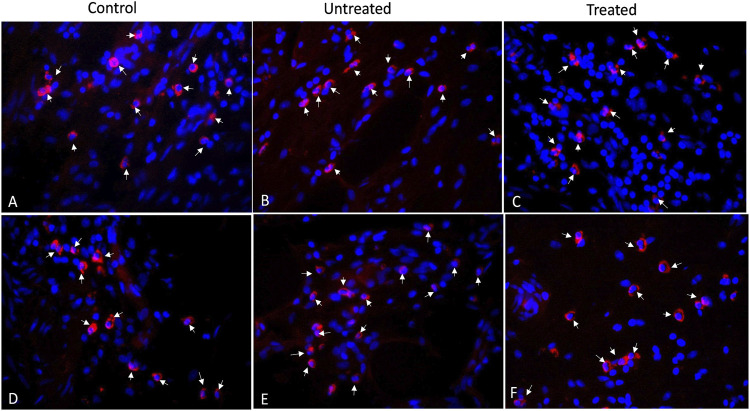
Immunofluorescence staining of CD3-positive lymphocytes **(A–C)** and CD68-positive macrophages **(D–F)** for control, untreated and treated diverticula (40X). CD3 and CD68 in red (arrows) and Nuclei in blue.

Figure 6 shows the histological images of CD3 and CD68 with fluorescence staining in control, untreated and treated diverticular lesions. CD3-positive lymphocytes and CD68-positive macrophages were mainly localized in the lamina propria and submucosal layer. The CD3 and CD68 infiltrated scores following 3 months of treatment are listed in Table 1. No significant differences in the inflammatory infiltrate of lymphocyte and macrophage were observed in the treated group as compared to the untreated and control groups.

## Discussion

Except for colectomy to remove diseased colon permanently, current leading managements for diverticular disease including medication and endoscopic/laparoscopic surgery cannot prevent the occurrence/recurrence of diverticulitis, bleeding and/or other complications (Cirocchi et al., 2015; Yamada et al., 2015; Kaushik et al., 2016; Longchamp et al., 2021). Developing more effective minimally invasive approaches leading to less complications and lower diverticulosis recurrence are urgently needed both for patients and the healthcare system. After investigating the biomechanical behaviors of the colon with the presence of a pouch-like structure created in swine (Guo et al., 2019), our group are the first to verify that stress distribution may change in diverticula where a vicious cycle may occur during the progress of diverticulosis (Patel et al., 2020). These findings provide a theoretical basis for a new therapeutic strategy by reducing local stress in diverticulum and improving the mechanical strength of the weakened colon wall. Our patch design is technically simple and feasible since it only requires a currently available biological tissue material and tissue adhesive that can be delivered using traditional laparoscopic techniques.

As a requirement for the proposed treatment, the mechanical properties of a biocompatible patch material should be such that it is stiff enough to prevent the pouch protrusion but still compliant enough to permit the natural motility of the colon. Xenogeneic elastin-based biomaterials such as porcine PVP and small intestinal submucosa (SIS) have been used for the repair of gastrointestinal tissue in animal studies (Kajitani et al., 2000; Tan et al., 2014). The PVP was deliberately chosen for the study because it contains abundant elastin and collagen with larger proportion of elastin than that in the primarily collagen-based biomaterials such as swine SIS or bovine pericardium. This structural character provides more mechanical compliance to the PVP as shown in our uniaxial testing of PVP, SIS, and bovine pericardium, where we confirmed that the incremental moduli in stress-strain curves and relaxation moduli in the Maxwell-Weichert model of PVP were approximately one-tenth of SIS and pericardium (Lu et al., 2022). In another mechanical test, we observed similar burst pressure between the PVP-constructed tubular prosthesis and rat artery tissue (386 mmHg vs. 329 mmHg), indicating the potential of the PVP as an arterial graft (Lu et al., 2019). With an *in vitro* mechanical testing and computational simulation (data are not shown), we further demonstrated that PVP from bovine is the optimal material for colon tissue reinforcement with an ideal mechanical compliance showing no observable effect on colonic deformation or motility. In addition, our group found the bovine PVP, after processing with glutaraldehyde, to be degradable over a 12-month period with low cytotoxicity, low immune rejection, and low inflammation, exhibiting improved physical and physiological characteristics for surgical implantation in rat, swine and dog models (Lu et al., 2019; Lu et al., 2020; Lu et al., 2022).

Cyanoacrylate-based tissue adhesives have been used in suture-less surgery in medical and veterinary applications since the 1970s (Amiel et al., 1999; Esposito et al., 2004; Miyano et al., 2004). Previous studies also identified tissue adhesives as the most promising colonic anastomotic sealants (Al-Mubarak and Al-Haddab., 2013; Vakalopoulos et al., 2017a). Histoacryl (an FDA-approved topical skin adhesive) was selected for the proposed patch application as it has shown good local tolerance and biocompatibility validated by extensive toxicological and implantation studies in human and animals (Kukleta et al., 2012). A recent animal study applying histoacryl on the colon for 28 days found that histoacryl maintained a strong adhesive bond to the tissue and induced a limited local host reaction without toxic effects on the bowel (Vakalopoulos et al., 2017b).

In this report, we performed a proof-of-concept study of adhesive patch treatment using our pig model of diverticulosis (Guo et al., 2019). To the best of our knowledge, this is the first study to utilize the Xenogeneic (bovine) biocompatible material to repair diverticular defects in a large animal model. We found the PVP patch to be easy to handle and apply onto the surface of colon. The use of histoacryl generated a strong bond between the patch and the colon with no need for additional suture fixation. After 3 months of follow-up, we found that 95% of pouch-like mucosa herniation was prevented from the colon wall. Furthermore, histological examination revealed a significant decrease in the diameter of the diverticular pouch over the 3-month implantation as compared to the untreated group. These results indicate that adhesive patch treatment may prevent diverticulosis progression or recurrence and accordingly reduce the chance of complications.

The use of the patch was safe, and no adverse events were identified. All experimental animals maintained their weights within the range of 5% of their pre-implantation weights during the observation period of 3 months. After the patch implantation, we observed no signs of colon infections, bleeding, perforation, stenosis or obstruction. Although slight focal adhesion between the large bowels occurred during the formation of diverticula in 3 CI-injected pigs, the patch application did not increase the severity and amount of intra-abdominal adhesions. Moreover, autonomic bowel peristalsis was noted *in vivo* in the treated colon segments of all animals. Those macroscopic findings reveal that the presence of PVP patch caused no visible adverse effects and did not seem to affect the functional bowel movement. The responses of colonic mechanical properties and functional movements to patch therapy require further investigations.

Microscopically, our histological examination showed that the damaged muscular layer on diverticulum was covered with the implanted material combined with growth of connective tissue. The representative multiphoton microscope imaging demonstrated that patch placement promoted repair of the damaged colon wall with newly growing muscle cells, collagen deposition and neovascularization in the muscle and serosa layers. Collagen, the main component of connective tissue, plays an important role in tissue repair and reconstruction (Krafts, 2010). Shogan et al. (2015) has reported that higher levels of collagen are associated with higher mechanical strength of intestine in a rat intestinal anastomoses model. In our study, 3 months of patch application resulted in a significantly increased levels of collagen as comparted to the untreated group, which possibly enhanced the mechanical strength of the diseased colon wall, and accordingly blocked the mucosa herniation. Lymphocytes and macrophages participate in the response of the adaptive immune system of the host (Vakalopoulos et al., 2017b). Although our assessment of the inflammatory reaction of the colon tissue to PVP patch displayed mild lymphocyte and macrophage infiltration, it was not significantly different from that of the untreated and control groups. This implies a normal histopathologic healing process of the colon wall following the treatment. Hence, we consider the PVP patch application a safe procedure. Longer observation time is necessary to evaluate the long-term behavior of the xenogeneic PVP application in swine.

With the rapid development of tissue engineering in regenerative medicine, developing biodegradable 3-dimensional scaffolds through the use of new biocompatible materials has been a growing research interest for tissue repair/regeneration (Choi et al., 2010; BaoLin and Ma, 2014). Synthetic prosthetic polymers, such as polyglycolic acid (PGA), polylactic acid (PLA), and polycaprolactone (PCL) were found to simulate the biologic and mechanical functions of the extracellular matrix (ECM), which could lead to tissue regrowing, remodeling, and response to injury (Timnak, et al., 2011; Sun et al., 2018). Moreover, composite scaffolds based on a combination of two or more materials (i.e., PCL-PHEA-PLA) have shown better mechanical strength and elasticity with a high level of cellular adhesion on fibers in animal studies (Lo Monte et al., 2012; Buscemi et al., 2017). These unique advantages are attractive as an alternative patch material for the treatment of injured colons that should be further explored in the future.

### Limitations of study

The current study mainly focuses on the feasibility of patch therapy for diverticulosis with a short-term observation period of up to 12 weeks. Therefore, this study provides no information about long-term safety and effectiveness, which is critical to assess the advantages of the proposed approach on the reduction of complications over time. Although the pig model was created by replicating the pathological process of diverticulosis, the chronic response of the colon to the PVP patch in swine may be different from that in human due to species differences and co-morbidities. Another limitation is the lack of colon mechanical testing to assess the impact of patch on bowel function. It is possible that patch implantation may alter colon mechanical properties, without causing visible changes in colon functional movement during the 12-week follow-up. Despite those limitations, this work may open a new way for researchers and clinicians to explore an effective preventive treatment for diverticulosis via mechanical reconstruction of the diseased colon.

## Conclusion

The adhesive PVP patch was used for the treatment of colon diverticulosis in a pig model. A 3-month patch implantation not only prevented mucosa herniation, but also accelerated tissue healing and strengthened colonic wall structure over the diverticular defects. The bovine PVP seems to be an excellent reinforcement biomaterial based on its efficacy, safety, stability, and ease of use. Our findings offer an alternative minimal invasive surgical procedure that may slow or even stop diverticulosis progression but maintain the anatomy and function of the colon. The current study demonstrated proof-of-concept using laparotomy, but future focus is needed to validate the feasibility of using traditional laparoscopy to deliver the PVP patch. Further studies are required to clarify the long-term benefits of this approach to minimize the risk of complications and recurrence of colonic diverticulosis.

## Data Availability

The original contributions presented in the study are included in the article/supplementary material, further inquiries can be directed to the corresponding author.
